# Supplementary material for: Combination prevention package of interventions for reducing vulnerability to HIV among adolescent girls and young women in Nigeria: An action research
Source: PLoS One. 2023 Jan 18;18(1):e0279077. doi: 10.1371/journal.pone.0279077 (PMC9847984; doi:10.1371/journal.pone.0279077)
Supplement: S1 File — (DOCX) [file pone.0279077.s001.docx]

**Combination prevention package of interventions for reducing vulnerability to HIV among adolescent girls and young women in Nigeria: An action research**

**AGYW Action research evaluation questionnaire**

**State Specific Interventions – Akwa-Ibom State**

| **No.** | **Questions and filters** | **Coding categories** | **Change idea explored** |
| --- | --- | --- | --- |
| 1601 | Have you ever been reached with any HIV prevention intervention in the past 6 months | Yes……….1  No………2 | Item Uwem/Babes alive/Cell meeting |
| 1601a | Please mention the HIV prevention intervention you were reached with in the last 6 months |  | Item Uwem/Babes alive/Cell meeting |
| 1602 | Did anyone discuss with you HIV prevention in the past 6 months | Yes.……….1  No………..2 | Item Uwem/Babes alive/Cell meeting |
| 1602a | Who discussed with you | Parent/guardian……….1  Health worker……….2  Peer……….3  Youth facilitator……….4  Others……….5 | Item Uwem/Babes alive/Cell meeting |
| 1602b | What HIV prevention/TREATMENT did they discuss with you | Condom use……….1  Abstinence……….2  HTS……….3  HIV Prevention……….4  STI Treatment……….5  HIV Treatment……….6 | Item Uwem/Babes alive/Cell meeting |
| 1603 | Has your father/mother/guardian communicated with you about HIV? | Yes.……….1  No………..2 | Item Uwem |
| 1603a | What did your father/mother/guardian communicated with you about HIV? | Condom use……….1  Abstinence……….2  HTS……….3  HIV Prevention……….4  STI Treatment……….5  HIV Treatment……….6 | Item Uwem |
| 1604 | Did your father/mother/guardian talk to you about menstruation and the effects of unplanned pregnancy in the past 6 months? | Yes………..1  No………..2 | Item Uwem |
| 1605 | Did your mother or caregiver in the last 6 months talk to you about sexually transmitted infections (STI), HIV? | Yes………..1  No………..2 | Item Uwem |
| 1606 | Did your mother or caregiver in the last 6 months talk to you on how to prevent HIV and STI in the last 6 months? | Yes………..1  No………..2 | Item Uwem |
| 1607 | Did your mother/caregiver tell you about people who spoke with her/him about pregnancy, HIV, STI and how to prevent HIV and STI in the last 6 months? | Yes………..1  No………..2 | Item Uwem |
| 1608 | Did your mother encourage you to attend an educational program about pregnancy, HIV/STI and how to prevent HIV/STI? | Yes………..1  No………..2 | Item Uwem |
| 1609 | Are you aware of or do you know any program in your community where HIV and STI information are discussed? | Yes………..1  No………..2 | AYP Cell Meetings |
| 1609a | Did you attend any program in your community were HIV and STI information were discussed with you? | Yes………..1  No………..2 | AYP Cell Meetings |
| 1609b | What were the things that you learnt in the meetings? | How to prevent HIV/STI…………1  How HIV/STI can be transmitted…..……2  How to manage STI………..3  How to set goals……….4  How to develop high self-esteem………..5  Condom negotiation…………6 | AYP Cell Meetings |
| 1610 | Did you attend any program in the last 2 months in your community were how to set goals, how to develop high self-esteem was discussed with you? | Yes………..1  No………..2 | AYP Cell Meetings |
| 1611 | Did you attend or hear of any programme where types of condoms and condom negotiation were taught? | Yes………..1  No………..2 | AYP Cell Meetings |
| 1612 | Do you know any place where you can get condoms in your community apart from the drug stores (chemist and pharmacy)? | Yes………..1  No………..2 | Stationary Condom Dispenser |
| 1612a | Can you mention some of those places where condoms can be collected free? | Salons……….1  Fashion shops………2  Tailoring shops……….3  Hair dressing salon………4  Viewing centres……..5  Others………6 | Stationary Condom Dispenser |
| 1612b | Have you collected condom in the last 6 months at any kind of shops such as Salon, tailoring shop, betNaija shops, sit-out, bars? | Yes………..1  No………..2 | Stationary Condom Dispenser |
| 1613 | Have you heard or attended any program were condom was distributed in your community in the last 6 months? | Yes………..1  No………..2 | Stationary Condom Dispenser |
| 1614 | Are you aware that a group was created where HIV and STI messages are being sent through WhatsApp? | Yes………..1  No………..2 | WhatsApp |
| 1614a | Are you among any group were HIV and STI messages were sent to you through Whatsapp? | Yes………..1  No………..2 | WhatsApp |
| 1615 | Are you aware of any young people’s program where people or you were counselled and tested for HIV in the last 6 months? | Yes………..1  No………..2 | Babes Alive |
| 1616 | Can you mention where HIV testing services can be accessed in your community? | Hospital….…….……1  Pharmacy…………...2  Maternity home……..3 | Babes Alive |
| 1617 | Do you know any place in your community where condom can be gotten anytime one desire it (where there is stationary condom dispenser)? | Yes………..1  No………..2 | Stationary Condom Dispenser |
| 1618a | Can you mention some of these places where the you can go to pick a free condom? |  | Gallant Dispenser |
| 1618b | Have you collected condom in the last 6 months at such place? | Yes………..1  No………..2 | Gallant Dispenser |
| 1619 | Are you aware of any condom distribution by any of your friends or any other young person in your community? | Yes………..1  No………..2 | Gallant Dispenser |
| 1619 | Have you collected free condom from any of your friends or any other young person in your community? | Yes………..1  No………..2 | Gallant Dispenser |

**State Specific Interventions – FCT Abuja**

| **No** | **Questions and Filters** | **Coding Categories** | **Change idea** |
| --- | --- | --- | --- |
| Q1601 | In the last 6 months has any of your friends talked about HIV message? | Yes……………………………….1  No……………………………….2 | Debunking the myths and misconceptions |
| Q1601a | If yes what is did he/she talk to you about | Condom use……….1  Abstinence……….2  HIV Testing Service……….3  HIV Prevention……….4  STI Treatment……….5  HIV Treatment……….6  Others…….7 | Debunking the myths and misconceptions |
| Q1602 | In the last 6 months has your friend invited you to any HIV program in your community? | Yes……………………………….1  No……………………………….2 | GF networking/Mentoring |
| Q1602a | What is the name of this program | Skills to health……….1  Abstinence……….2  Others……….3 | GF networking/Mentoring |
| Q1602c | What were you taught in the meeting | Condom use……….1  Abstinence……….2  HIV Testing Service……….3  HIV Prevention……….4  STI Treatment……….5  Others…….6 | GF networking/Mentoring |
| Q1603 | In the last 6 months have you been invited for HIV test through a friend referral in your community? | Yes……………………………….1  No……………………………….2 | GF networking |
| Q1604 | In the last 6 months have you been tested for HIV through your friend’s referral? | Yes……………………………….1  No……………………………….2 | GF networking |
| Q1603a | In the last 6 months Have you accessed STI treatment through an outreach or referred to a hospital in your community? | Yes……………………………….1  No……………………………….2 | Skills to Health |
| Q1603b | In the last 6 months, has any of your friends informed you about STI message? | Yes……………………………….1  No……………………………….2 | GF networking/Mentoring |
| Q1603c | In the last 6 months have you received free condom from your friend? | Yes……………………………….1  No……………………………….2 | Condom use for us |
| Q1604a | In the last 6 months, has your sexual partner discussed correct and consistent condom use with you? | Yes……………………………….1  No……………………………….2  No Sexual Partner……………………………….3 | Condom use for us |
| Q1604c | In the last 6 months, have you ever collected free condoms from your sexual partner or friend? | Yes……………………………….1  No……………………………….2 | Condom use for us |
| Q1605a | In the last 6 months, has your mother ever talked to you about Sex, HIV and unintended pregnancy? | Yes……………………………….1  No……………………………….2 | My daughter my pride |
| Q1605b | Has your parent/guardian ever invited you for an HIV program? | Yes……………………………….1  No……………………………….2 | My daughter my pride |
| Q1605c | In the last 2 months have you become more comfortable discussing your health and wellbeing? | Yes……………………………….1  No……………………………….2 | My daughter my pride |

*Debunking HIV misconceptions already covered in the original question

**State Specific Interventions – Kaduna State**

| **No.** | **Questions and filters** | **Coding categories** | **Change topic / Change idea** |
| --- | --- | --- | --- |
| **Q1601** | Has any of your parents discussed anything about sex with you in the last 6 months | Yes……….1  No………2 | M2D |
| **Q1601a** | Who discussed with you | Mother……….1  Father……….2  Both.……….3 | M2D and Father of AYPs |
| **Q1601b** | Did this mother/Father tell you about any danger of having sex early? | Yes.……….1  No………..2 | M2D and Father of AYPs |
| **Q1601c** | What dangers of having sex early in life did mother/Father tell you about? | STI’s…………1  HIV/AIDS…………2  Pregnancy…………3  Loss of educational opportunity………....4  Psychological problems…………5  Others …………6 | M2D and Father of AYPs |
| **Q1601d** | Did your mother/Father tell you anything about HIV/AIDS transmission (or how it is spread) | Yes.……….1  No………..2 | M2D and Father of AYPs |
| **Q1601e** | What did your mother/Father tell you about HIV/AIDS transmission?  (allow for multiple response) | Gotten from unprotected sexual activity……….1  Gotten from sharing of sharp objects……….2  Gotten from infected mother to her child……….3  Unsafe blood transfusion……….4  Others...……..5 | M2D and Father of AYPs |
| **Q1601f** | Did your mother/Father tell you anything about HIV/AIDS prevention | Yes.……….1  No………..2 | M2D and Father of AYPs |
| **Q1601g** | What HIV prevention method did they discuss with you | Condom use……….1  Abstinence……….2  HTS……….3  Being faithful to one partner………4 | M2D and Father of AYPs |
| **Q1602** | Has any of your friends told anything about HIV/AIDS in the last 6 months | Yes ………...1  No………….2 | Tell a friend |
| **Q1603** | What did they tell you about HIV/AIDS | Prevention…………..1  Transmission…………..2  HTS..…………3  PMTCT………..…4 | TAF |
| **Q1604** | At what age do you think a lady should get married | …………………… | M2D |
| **Q1605** | Has anyone in your family discussed the dangers of early marriage with you in the last 6 months | Yes.……….1  No………..2 | M2D and Father of AYPs |
| **Q1605a** | What were the dangers of early marriage discussed with you | STI’s…………1  HIV/AIDS…………2  Complicated Pregnancy…………3  Loss of educational opportunity………....4  Psychological problems…………5  Gender based violence ………….6  Others …………7 | M2D and Father of AYPs |
| **Q1606** | Will you have support from your mother if you don’t want to marry early? | Yes.……….1  No………..2 | M2D |
| **Q1607** | Will you have support from your father if you don’t want to marry early? | Yes.……….1  No………..2 | Father of AYPs |
| **Q1608** | Has anyone in your community leadership discussed premarital HIV counselling and testing with you in the last 6 months | Yes.……….1  No………..2 | Traditional / community leaders |
| **Q1609** | Who discussed with you | ………………………. | Traditional / community leaders |
| **Q1610** | Should married women who develop STI inform their husbands | Yes.……….1  No………..2 | Husbands of married AYPs |
| **Q1611** | Do you think Husbands of married women who develop a STI will agree to go for check up | Yes.……….1  No………..2 | Husbands of married AYPs |
| **Q1612** | Will your husband go for testing and treatment with you if you have a STI? (married AYPs only) | Yes.……….1  No………..2 | Husbands of married AYPs |
| **Q1613** | Have you had an STI in the last 6 months | Yes.……….1  No………..2 | Husbands of married AYPs |
| **Q1613a** | Did you go for treatment for the STI | Yes.……….1  No………..2 | Husbands of married AYPs |
| **Q1613b** | Did your Husband go for the treatment? | Yes.……….1  No………..2 | Husbands of married AYPs |

**State Specific Interventions – Oyo State**

| **No.** | **Questions and filters** | **Coding categories** | **Change idea explored** |
| --- | --- | --- | --- |
| **Q1601** | Did anyone discuss with you HIV prevention in the past 6 months | Yes.……….1  No………..2 |  |
| **Q1601a** | Who discussed with you | Parent/guardian……….1  Health worker……….2  Youth facilitator……….3  Others……….3 |  |
| **Q1601b** | What HIV prevention/TREATMENT did they discuss with you (tick all that apply) | Condom use……….1  Abstinence……….2  HTS……….3  HIV Prevention……….4  STI Treatment……….5  Stigma and discrimination……….6 |  |
| **Q1602** | Have you heard about any programme in your community where HIV,HTS and SRH were discussed? | Yes.……….1  No………..2 | Social to Health (S2H) |
| **Q1602b** | Did you attend any programme in your community where HIV and HTS were discussed in the last 6 months? | Yes.……….1  No………..2 | Social to Health (S2H) |
| **Q1603** | Have you received HIV counselling and tested for HIV/AIDS in the last 6 months? | Yes.……….1  No………..2 | Social to Health (S2H) |
| **Q1604** | Are you aware that a group was created where HIV and other SRH messages are being sent? | Yes.……….1  No………..2 | Social to Health (S2H) |
| **Q1605** | Are you among any group where HIV and other SRH messages were sent to you through whatsapp? | Yes.……….1  No………..2 | Online session via Whatsapp |
| **Q1605a** | How often do you check the messages | Often…….1  Rarely…...2  Not at all…3 | Online session via Whatsapp |
| **Q1606** | Has any of your friend/peer talked with you about condom and condom use? | Yes.……….1  No………..2 | Condom distribution via peer to peer |
| **Q1606a** | What did your friend/peer talk with you about condom and condom use? |  | Condom distribution via peer to peer |
| **Q1607** | Has any of your friend/ peer communicated to you about where to get condom? | Yes.……….1  No………..2 | Condom distribution via peer to peer |
| **Q1607a** | Can you mention any of these places? |  | Condom distribution via peer to peer |
| **Q1607b** | Have you ever gone to pick condom from this place? | Yes.……….1  No………..2 | Condom distribution via peer to peer |
| **Q1608** | Are you aware of any programme/ outreach where HIV testing is being conducted in your community? | Yes.……….1  No………..2 | Social to Health/STI Outreach |
| **Q1609** | Have you being to a HIV testing outreach in your community in the last 6 months? | Yes.……….1  No………..2 | HTS Outreach |
| **Q1609a** | How often is the HTS outreach conducted in your community? | Very often……1  Rarely……….2  Not at all…….3 | HTS Outreach |
| **Q1610** | Do people come to your community for free HTS? | Yes.……….1  No………..2 | HTS Outreach |
| **Q1611** | Can you mention where HTS can be accessed in your community? |  | HTS Outreach |
| **Q1612** | Have you participated in any activity related to sexual infection, Stigma and discrimination, and gender based violence including rape in the last 6 months? | Yes.……….1  No………..2 | IPC with AYP via snowballing |
| **Q1612a** | Can you mention what the message was all about? |  | IPC with AYP via snowballing |
| **Q1613** | Are you aware of any STI services being provided in your community? | Yes.……….1  No………..2 | STI Outreaches |
| **Q1613a** | Have you accessed any STI services being provided in your community? | Yes.……….1  No………..2 | STI Outreaches |
