# Supplementary material for: Combination prevention package of interventions for reducing vulnerability to HIV among adolescent girls and young women in Nigeria: An action research
Source: PLoS One. 2023 Jan 18;18(1):e0279077. doi: 10.1371/journal.pone.0279077 (PMC9847984; doi:10.1371/journal.pone.0279077)
Supplement: S2 File — (DOCX) [file pone.0279077.s002.docx]

**Combination prevention package of interventions for reducing vulnerability to HIV among adolescent girls and young women in Nigeria: An action research**

# FGD with Females Adolescents and Young Persons

**Introduction:** My name is…… ……… I am working for the State Government through the Ministry of Health and SACA. We are carrying out a study on young people here in [**NAME OF CITY, TOWN OR SITE,**] on behalf of the government of Nigeria, in order to find out about certain behaviours that affect young women’s health in this environment.

**Confidentiality and consent:** I am going to ask you questions some of which may be very personal. Your answers are completely confidential. Your name will not be written on this form, and will never be used in connection with any of the information you tell me. You may need to know that this exercise is taking place in at least 3 other states of the country. Your honest answers during our discussions will help us better understand what people think, say and do about certain kinds of behaviours. The information collected from you and people like you will help the government to find solutions to some health problems affecting young people in this environment. We would greatly appreciate your help in participating in this interview. You may discontinue with the interview at any stage that you may wish to and there will be no penalties for doing this. I also wish to inform you that we will like to tape record our discussion to make the process fast. The recorded information will however be kept strictly confidential.

1. **Socio-Demographic characteristics of FGD participants**

**LGA _____________ Community Name ____________________ Date _________**

**Name of FGD Group_____________________________________**

| **No** | **Age** | **Marital Status** | **Gender** | **Religion** | **Ethnic group** | **Level of Education** | **Years of Residence in the community** |
| --- | --- | --- | --- | --- | --- | --- | --- |
| **P1** |  |  |  |  |  |  |  |
| **P2** |  |  |  |  |  |  |  |
| **P3** |  |  |  |  |  |  |  |
| **P4** |  |  |  |  |  |  |  |
| **P5** |  |  |  |  |  |  |  |
| **P6** |  |  |  |  |  |  |  |
| **P7** |  |  |  |  |  |  |  |
| **P8** |  |  |  |  |  |  |  |
| **P9** |  |  |  |  |  |  |  |
| **P10** |  |  |  |  |  |  |  |

1. What are the general *health* problems among young people in this community?
2. What *reproductive health* (sexuality-related issues) challenges are common among young people? Probe for STIs including HIV/AIDS if not mentioned.

Probe for specific challenges confronting young women and girls? Why are these challenges peculiar to young women and girls?

1. What do you think are the causes of these *reproductive health* problems confronting young people? Please ask this question for each problem identified.
2. How will you describe the risks of young women and girls to HIV/AIDS infection in your community?
3. Who do you think is more at risk between young men and young women? Why do you think so?
4. Why/in what situations are women and girls more at risk of acquiring HIV/AIDS infection in this community?
5. Mention different means of acquiring HIV/AIDS and other STIs
6. Mention some ways of preventing HIV infections in your community.
7. Mention some sources of information for young people on reproductive health in your community? (probe for information on menstruation, unplanned pregnancy, STI, unprotected sex, HTS uptake etc.)
8. Also probe for the kind of information that young people get from each of the sources mentioned in question 10 (probe for information from father, mother, peers if not mentioned)
9. Which of these sources of information do you think are more effective?
10. What do young people do to protect themselves against STIs, unplanned pregnancy and HIV/AIDS in this community? (Probe for use of condom and where they get it, HTS and HIV care if not mentioned)
11. If there is an HIV positive young person in this community, what will be the reactions of the community members? Please probe for what will be the reaction of the respondents?

**FOR LGAs WHERE INTERVENTION TOOK PLACE**

1. In the last 6 months what intervention programmes on HIV/AIDS are you aware of in this community?
2. Probe for specific benefits of each of the intervention programme mentioned for young people. Also probe for problems they have or foresee with the programmes
3. Mention some reproductive health services that are still not easily accessible to AYP? Probe for the barriers for accessing such services

**FOR CONTROL LGA**

1. Mention some reproductive health services that are not easily accessible to AYP? Probe for the barriers for accessing such services
2. What other information do you think may be useful for this study?

# ACTION RESEARCH FOR ADOLESCENTS AND YOUNG PERSONS IN SELECTED STATES OF NIGERIA

# FGD with Young and Older Men

**Introduction:** My name is…… ……… I am working for the State Government through the Ministry of Health and SACA. We are carrying out a study on young people here in [**NAME OF CITY, TOWN OR SITE,**] on behalf of the government of Nigeria, in order to find out about certain behaviours that affect young women’s health in this environment.

**Confidentiality and consent:** I am going to ask you questions some of which may be very personal. Your answers are completely confidential. Your name will not be written on this form, and will never be used in connection with any of the information you tell me. You may need to know that this exercise is taking place in at least 3 other states of the country. Your honest answers during our discussions will help us better understand what people think, say and do about certain kinds of behaviours. The information collected from you and people like you will help the government to find solutions to some health problems affecting young people in this environment. We would greatly appreciate your help in participating in this interview. You may discontinue with the interview at any stage that you may wish to and there will be no penalties for doing this. I also wish to inform you that we will like to tape record our discussion to make the process fast. The recorded information will however be kept strictly confidential.

1. **Socio-Demographic characteristics of FGD participants**

**LGA _____________ Community Name ____________________ Date _________**

**Name of FGD Group_____________________________________**

| **No** | **Age** | **Marital Status** | **Gender** | **Religion** | **Ethnic group** | **Level of Education** | **Years of Residence in the community** |
| --- | --- | --- | --- | --- | --- | --- | --- |
| **P1** |  |  |  |  |  |  |  |
| **P2** |  |  |  |  |  |  |  |
| **P3** |  |  |  |  |  |  |  |
| **P4** |  |  |  |  |  |  |  |
| **P5** |  |  |  |  |  |  |  |
| **P6** |  |  |  |  |  |  |  |
| **P7** |  |  |  |  |  |  |  |
| **P8** |  |  |  |  |  |  |  |
| **P9** |  |  |  |  |  |  |  |
| **P10** |  |  |  |  |  |  |  |

1. What are the general *health* problems among young people in this community?
2. What *reproductive health* (sexuality-related issues) challenges are common among young people? Probe for STIs including HIV/AIDS if not mentioned.

Probe for specific challenges confronting young women and girls? Why are these challenges peculiar to young women and girls?

1. What do you think are the causes of these *reproductive health* problems confronting young people? Please ask this question for each problem identified.
2. How will you describe the risks of young women and girls to HIV/AIDS infection in your community?
3. Who do you think is more at risk between young men and young women? Why do you think so?
4. Why/in what situations are women and girls more at risk of acquiring HIV/AIDS infection in this community?
5. Mention different means of acquiring HIV/AIDS and other STIs
6. Mention some ways of preventing HIV infections in your community.
7. Mention some sources of information for young people on reproductive health in your community? (Probe for information on menstruation, unplanned pregnancy, STI, unprotected sex, HTS uptake etc.)
8. Also probe for the kind of information that young people get from each of the sources mentioned in question 10 (probe for information from father, mother, peers if not mentioned)
9. Which of these sources of information do you think are more effective?
10. What do young people do to protect themselves against STIs, unplanned pregnancy and HIV/AIDS in this community? (Probe for use of condom and where they get it, HTS and HIV care if not mentioned)
11. If there is an HIV positive young person in this community, what will be the reactions of the community members? Please probe for what will be the reaction of the respondents?
12. What are the roles of men in reducing the spread of HIV in the community?

**FOR LGAs WHERE INTERVENTION TOOK PLACE**

1. In the last 6 months what intervention programmes on HIV/AIDS are you aware of in this community?
2. Probe for specific benefits of each of the intervention programme mentioned for young people. Also probe for problems they have or foresee with the programmes
3. Mention some reproductive health services that are still not easily accessible to AYP? Probe for the barriers for accessing such services

**FOR CONTROL LGA**

1. Mention some reproductive health services that are not easily accessible to AYP? Probe for the barriers for accessing such services
2. What other information do you think may be useful for this study?

# ACTION RESEARCH FOR ADOLESCENTS AND YOUNG PERSONS IN SELECTED STATES OF NIGERIA

# FGD with Adolescents’ mothers/caregivers

**Introduction:** My name is…… ……… I am working for the State Government through the Ministry of Health and SACA. We are carrying out a study on young people here in [**NAME OF CITY, TOWN OR SITE,**] on behalf of the government of Nigeria, in order to find out about certain behaviours that affect young women’s health in this environment.

**Confidentiality and consent:** I am going to ask you questions some of which may be very personal. Your answers are completely confidential. Your name will not be written on this form, and will never be used in connection with any of the information you tell me. You may need to know that this exercise is taking place in at least 3 other states of the country. Your honest answers during our discussions will help us better understand what people think, say and do about certain kinds of behaviours. The information collected from you and people like you will help the government to find solutions to some health problems affecting young people in this environment. We would greatly appreciate your help in participating in this interview. You may discontinue with the interview at any stage that you may wish to and there will be no penalties for doing this. I also wish to inform you that we will like to tape record our discussion to make the process fast. The recorded information will however be kept strictly confidential.

1. **Socio-Demographic characteristics of FGD participants**

**LGA _____________ Community Name ____________________ Date _________**

**Name of FGD Group_____________________________________**

| **No** | **Age** | **Marital Status** | **Gender** | **Religion** | **Ethnic group** | **Level of Education** | **Years of Residence in the community** |
| --- | --- | --- | --- | --- | --- | --- | --- |
| **P1** |  |  |  |  |  |  |  |
| **P2** |  |  |  |  |  |  |  |
| **P3** |  |  |  |  |  |  |  |
| **P4** |  |  |  |  |  |  |  |
| **P5** |  |  |  |  |  |  |  |
| **P6** |  |  |  |  |  |  |  |
| **P7** |  |  |  |  |  |  |  |
| **P8** |  |  |  |  |  |  |  |
| **P9** |  |  |  |  |  |  |  |
| **P10** |  |  |  |  |  |  |  |

1. What are the general *health* problems among young people in this community?
2. What *reproductive health* (sexuality-related issues) challenges are common among young people? Probe for STIs including HIV/AIDS if not mentioned.

Probe for specific challenges confronting young women and girls? Why are these challenges peculiar to young women and girls?

1. What do you think are the causes of these *reproductive health* problems confronting young people? Please ask this question for each problem identified.
2. How will you describe the risks of young women and girls to HIV/AIDS infection in your community?
3. Who do you think is more at risk between young men and young women? Why do you think so?
4. Why/in what situations are women and girls more at risk of acquiring HIV/AIDS infection in this community?
5. Mention different means of acquiring HIV/AIDS and other STIs
6. Mention some ways of preventing HIV infections in your community.
7. Mention some sources of information for young people on reproductive health in your community? (probe for information on menstruation, unplanned pregnancy, STI, unprotected sex, HTS uptake etc.)
8. Also probe for the kind of information that young people get from each of the sources mentioned in question 10 (probe for information from father, mother, peers if not mentioned)
9. Which of these sources of information do you think are more effective?
10. What do young people do to protect themselves against STIs, unplanned pregnancy and HIV/AIDS in this community? (Probe for use of condom and where they get it, HTS and HIV care if not mentioned)
11. If there is an HIV positive young person in this community, what will be the reactions of the community members? Please probe for what will be the reaction of the respondents?
12. What are the roles of adult women/caregivers in reducing the spread of HIV in the community?

**FOR LGAs WHERE INTERVENTION TOOK PLACE**

1. In the last 6 months what intervention programmes on HIV/AIDS are you aware of in this community?
2. Probe for specific benefits of each of the intervention programme mentioned for young people. Also probe for problems they have or foresee with the programmes
3. Mention some reproductive health services that are still not easily accessible to AYP? Probe for the barriers for accessing such services

**FOR CONTROL LGA**

1. Mention some reproductive health services that are not easily accessible to AYP? Probe for the barriers for accessing such services
2. What other information do you think may be useful for this study?

# ACTION RESEARCH FOR ADOLESCENTS AND YOUNG PERSONS IN SELECTED STATES OF NIGERIA

# IDI Guide for Religious leaders

**Introduction:** My name is…… ……… I am working for the State Government through the Ministry of Health and SACA. We are carrying out a study on young people here in [**NAME OF CITY, TOWN OR SITE,**] on behalf of the government of Nigeria, in order to find out about certain behaviours that affect young women’s health in this environment.

**Confidentiality and consent:** I am going to ask you questions some of which may be very personal. Your answers are completely confidential. Your name will not be written on this form, and will never be used in connection with any of the information you tell me. You may need to know that this exercise is taking place in at least 3 other states of the country. Your honest answers during our discussions will help us better understand what people think, say and do about certain kinds of behaviours. The information collected from you and people like you will help the government to find solutions to some health problems affecting young people in this environment. We would greatly appreciate your help in participating in this interview. You may discontinue with the interview at any stage that you may wish to and there will be no penalties for doing this. I also wish to inform you that we will like to tape record our discussion to make the process fast. The recorded information will however be kept strictly confidential.

1. Socio-Demographic characteristics of respondent

- Age
- Sex
- Religion
- Educational level

1. What are the general *health* problems among young people in this community?
2. What *reproductive health* (sexuality-related issues) challenges are common among young people? Probe for STIs including HIV/AIDS if not mentioned.

Probe for specific challenges confronting young women and girls? Why are these challenges peculiar to young women and girls?

1. What do you think are the causes of these *reproductive health* problems confronting young people? Please ask this question for each problem identified.
2. How will you describe the risks of young women and girls to HIV/AIDS infection in your community?
3. Who do you think is more at risk between young men and young women? Why do you think so?
4. Why/in what situations are women and girls more at risk of acquiring HIV/AIDS infection in this community?
5. Mention different means of acquiring HIV/AIDS and other STIs
6. Mention some ways of preventing HIV infections in your community.
7. Mention some sources of information for young people on reproductive health in your community? (probe for information on menstruation, unplanned pregnancy, STI, unprotected sex, HTS uptake etc.)
8. Also probe for the kind of information that young people get from each of the sources mentioned in question 10 (probe for information from father, mother, peers if not mentioned)
9. Which of these sources of information do you think are more effective?
10. What do young people do to protect themselves against STIs, unplanned pregnancy and HIV/AIDS in this community? (Probe for use of condom and where they get it, HTS and HIV care if not mentioned)
11. If there is an HIV positive young person in this community, what will be the reactions of the community members? Please probe for what will be the reaction of the respondents?
12. What are the roles of religious leaders/organisation in reducing the spread of HIV in the community

**FOR LGAs WHERE INTERVENTION TOOK PLACE**

1. In the last 6 months what intervention programmes on HIV/AIDS are you aware of in this community?
2. Probe for specific benefits of each of the intervention programme mentioned for young people. Also probe for problems they have or foresee with the programmes
3. Mention some reproductive health services that are still not easily accessible to AYP? Probe for the barriers for accessing such services

**FOR CONTROL LGA**

1. Mention some reproductive health services that are not easily accessible to AYP? Probe for the barriers for accessing such services
2. What other information do you think may be useful for this study?

# ACTION RESEARCH FOR ADOLESCENTS AND YOUNG PERSONS IN SELECTED STATES OF NIGERIA

# IDI Guide for Traditional leaders

**Introduction:** My name is…… ……… I am working for the State Government through the Ministry of Health and SACA. We are carrying out a study on young people here in [**NAME OF CITY, TOWN OR SITE,**] on behalf of the government of Nigeria, in order to find out about certain behaviours that affect young women’s health in this environment.

**Confidentiality and consent:** I am going to ask you questions some of which may be very personal. Your answers are completely confidential. Your name will not be written on this form, and will never be used in connection with any of the information you tell me. You may need to know that this exercise is taking place in at least 3 other states of the country. Your honest answers during our discussions will help us better understand what people think, say and do about certain kinds of behaviours. The information collected from you and people like you will help the government to find solutions to some health problems affecting young people in this environment. We would greatly appreciate your help in participating in this interview. You may discontinue with the interview at any stage that you may wish to and there will be no penalties for doing this. I also wish to inform you that we will like to tape record our discussion to make the process fast. The recorded information will however be kept strictly confidential.

1. Socio-Demographic characteristics of respondent

- Age
- Sex
- Religion
- Educational level

1. What are the general *health* problems among young people in this community?
2. What *reproductive health* (sexuality-related issues) challenges are common among young people? Probe for STIs including HIV/AIDS if not mentioned.

Probe for specific challenges confronting young women and girls? Why are these challenges peculiar to young women and girls?

1. What do you think are the causes of these *reproductive health* problems confronting young people? Please ask this question for each problem identified.
2. How will you describe the risks of young women and girls to HIV/AIDS infection in your community?
3. Who do you think is more at risk between young men and young women? Why do you think so?
4. Why/in what situations are women and girls more at risk of acquiring HIV/AIDS infection in this community?
5. Mention different means of acquiring HIV/AIDS and other STIs
6. Mention some ways of preventing HIV infections in your community.
7. Mention some sources of information for young people on reproductive health in your community? (probe for information on menstruation, unplanned pregnancy, STI, unprotected sex, HTS uptake etc.)
8. Also probe for the kind of information that young people get from each of the sources mentioned in question 10 (probe for information from father, mother, peers if not mentioned)
9. Which of these sources of information do you think are more effective?
10. What do young people do to protect themselves against STIs, unplanned pregnancy and HIV/AIDS in this community? (Probe for use of condom and where they get it, HTS and HIV care if not mentioned)
11. If there is an HIV positive young person in this community, what will be the reactions of the community members? Please probe for what will be the reaction of the respondents?
12. What are the roles of traditional leaders/institutions in reducing the spread of HIV in the community

**FOR LGAs WHERE INTERVENTION TOOK PLACE**

1. In the last 6 months what intervention programmes on HIV/AIDS are you aware of in this community?
2. Probe for specific benefits of each of the intervention programme mentioned for young people. Also probe for problems they have or foresee with the programmes
3. Mention some reproductive health services that are still not easily accessible to AYP? Probe for the barriers for accessing such services

**FOR CONTROL LGA**

1. Mention some reproductive health services that are not easily accessible to AYP? Probe for the barriers for accessing such services
2. What other information do you think may be useful for this study?

# ACTION RESEARCH FOR ADOLESCENTS AND YOUNG PERSONS IN SELECTED STATES OF NIGERIA

# IDI Guide for Youth leaders

**Introduction:** My name is…… ……… I am working for the State Government through the Ministry of Health and SACA. We are carrying out a study on young people here in [**NAME OF CITY, TOWN OR SITE,**] on behalf of the government of Nigeria, in order to find out about certain behaviours that affect young women’s health in this environment.

**Confidentiality and consent:** I am going to ask you questions some of which may be very personal. Your answers are completely confidential. Your name will not be written on this form, and will never be used in connection with any of the information you tell me. You may need to know that this exercise is taking place in at least 3 other states of the country. Your honest answers during our discussions will help us better understand what people think, say and do about certain kinds of behaviours. The information collected from you and people like you will help the government to find solutions to some health problems affecting young people in this environment. We would greatly appreciate your help in participating in this interview. You may discontinue with the interview at any stage that you may wish to and there will be no penalties for doing this. I also wish to inform you that we will like to tape record our discussion to make the process fast. The recorded information will however be kept strictly confidential.

1. Socio-Demographic characteristics of respondent

- Age
- Sex
- Religion
- Educational level

1. What are the general *health* problems among young people in this community?
2. What *reproductive health* (sexuality-related issues) challenges are common among young people? Probe for STIs including HIV/AIDS if not mentioned.

Probe for specific challenges confronting young women and girls? Why are these challenges peculiar to young women and girls?

1. What do you think are the causes of these *reproductive health* problems confronting young people? Please ask this question for each problem identified.
2. How will you describe the risks of young women and girls to HIV/AIDS infection in your community?
3. Who do you think is more at risk between young men and young women? Why do you think so?
4. Why/in what situations are women and girls more at risk of acquiring HIV/AIDS infection in this community?
5. Mention different means of acquiring HIV/AIDS and other STIs
6. Mention some ways of preventing HIV infections in your community.
7. Mention some sources of information for young people on reproductive health in your community? (probe for information on menstruation, unplanned pregnancy, STI, unprotected sex, HTS uptake etc.)
8. Also probe for the kind of information that young people get from each of the sources mentioned in question 10 (probe for information from father, mother, peers if not mentioned)
9. Which of these sources of information do you think are more effective?
10. What do young people do to protect themselves against STIs, unplanned pregnancy and HIV/AIDS in this community? (Probe for use of condom and where they get it, HTS and HIV care if not mentioned)
11. If there is an HIV positive young person in this community, what will be the reactions of the community members? Please probe for what will be the reaction of the respondents?
12. What are the roles of youth leaders/organisation in reducing the spread of HIV in the community

**FOR LGAs WHERE INTERVENTION TOOK PLACE**

1. In the last 6 months what intervention programmes on HIV/AIDS are you aware of in this community?
2. Probe for specific benefits of each of the intervention programme mentioned for young people. Also probe for problems they have or foresee with the programmes
3. Mention some reproductive health services that are still not easily accessible to AYP? Probe for the barriers for accessing such services

**FOR CONTROL LGA**

1. Mention some reproductive health services that are not easily accessible to AYP? Probe for the barriers for accessing such services
2. What other information do you think may be useful for this study?

# ACTION RESEARCH FOR ADOLESCENTS AND YOUNG PERSONS IN SELECTED STATES OF NIGERIA

# KII Guide for LGA level HIV program desk officers and LGA level health workers

**Introduction:** My name is…… ……… I am working for the State Government through the Ministry of Health and SACA. We are carrying out a study on young people here in [**NAME OF CITY, TOWN OR SITE,**] on behalf of the government of Nigeria, in order to find out about certain behaviours that affect young women’s health in this environment.

**Confidentiality and consent:** I am going to ask you questions some of which may be very personal. Your answers are completely confidential. Your name will not be written on this form, and will never be used in connection with any of the information you tell me. You may need to know that this exercise is taking place in at least 3 other states of the country. Your honest answers during our discussions will help us better understand what people think, say and do about certain kinds of behaviours. The information collected from you and people like you will help the government to find solutions to some health problems affecting young people in this environment. We would greatly appreciate your help in participating in this interview. You may discontinue with the interview at any stage that you may wish to and there will be no penalties for doing this. I also wish to inform you that we will like to tape record our discussion to make the process fast. The recorded information will however be kept strictly confidential.

1. Socio-Demographic characteristics of respondent

- Age
- Sex
- Religion
- Educational level

1. What are the general *health* problems among young people in this community?
2. What *reproductive health* (sexuality-related issues) challenges are common among young people? Probe for STIs including HIV/AIDS if not mentioned.

Probe for specific challenges confronting young women and girls? Why are these challenges peculiar to young women and girls?

1. What do you think are the causes of these *reproductive health* problems confronting young people? Please ask this question for each problem identified.
2. How will you describe the risks of young women and girls to HIV/AIDS infection in your community?
3. Who do you think is more at risk between young men and young women? Why do you think so?
4. Why/in what situations are women and girls more at risk of acquiring HIV/AIDS infection in this community?
5. Mention different means of acquiring HIV/AIDS and other STIs
6. Mention some ways of preventing HIV infections in your community.
7. Mention some sources of information for young people on reproductive health in your community? (probe for information on menstruation, unplanned pregnancy, STI, unprotected sex, HTS uptake etc.)
8. Also probe for the kind of information that young people get from each of the sources mentioned in question 10 (probe for information from father, mother, peers if not mentioned)
9. Which of these sources of information do you think are more effective?
10. What do young people do to protect themselves against STIs, unplanned pregnancy and HIV/AIDS in this community? (Probe for use of condom and where they get it, HTS and HIV care if not mentioned)
11. If there is an HIV positive young person in this community, what will be the reactions of the community members?

**Q 15 – 17 ARE FOR LGAs WHERE INTERVENTION TOOK PLACE**

1. In the last 6 months what intervention programmes on HIV/AIDS are you aware of in this community?
2. Probe for specific benefits of each of the intervention programme mentioned for young people. Also probe for problems they have or foresee with the programmes
3. Mention some reproductive health services that are still not easily accessible to AYP? Probe for the barriers for accessing such services

**Q 18 is FOR CONTROL LGA**

1. Mention some reproductive health services that are not easily accessible to AYP? Probe for the barriers for accessing such services
2. What other information do you think may be useful for this study?

# ACTION RESEARCH FOR ADOLESCENTS AND YOUNG PERSONS IN SELECTED STATES OF NIGERIA

# KII Guide for Women Affairs Desk Officer

**Introduction:** My name is…… ……… I am working for the State Government through the Ministry of Health and SACA. We are carrying out a study on young people here in [**NAME OF CITY, TOWN OR SITE,**] on behalf of the government of Nigeria, in order to find out about certain behaviours that affect young women’s health in this environment.

**Confidentiality and consent:** I am going to ask you questions some of which may be very personal. Your answers are completely confidential. Your name will not be written on this form, and will never be used in connection with any of the information you tell me. You may need to know that this exercise is taking place in at least 3 other states of the country. Your honest answers during our discussions will help us better understand what people think, say and do about certain kinds of behaviours. The information collected from you and people like you will help the government to find solutions to some health problems affecting young people in this environment. We would greatly appreciate your help in participating in this interview. You may discontinue with the interview at any stage that you may wish to and there will be no penalties for doing this. I also wish to inform you that we will like to tape record our discussion to make the process fast. The recorded information will however be kept strictly confidential.

1. Socio-Demographic characteristics of respondent

Age

Sex

Religion

Educational level

1. What are the general *health* problems among young people in this community?
2. What *reproductive health* (sexuality-related issues) challenges are common among young people? Probe for STIs including HIV/AIDS if not mentioned.

Probe for specific challenges confronting young women and girls? Why are these challenges peculiar to young women and girls?

1. What do you think are the causes of these *reproductive health* problems confronting young people? Please ask this question for each problem identified.
2. How will you describe the risks of young women and girls to HIV/AIDS infection in your community?
3. Who do you think is more at risk between young men and young women? Why do you think so?
4. Why/in what situations are women and girls more at risk of acquiring HIV/AIDS infection in this community?
5. Mention different means of acquiring HIV/AIDS and other STIs
6. Mention some ways of preventing HIV infections in your community.
7. Mention some sources of information for young people on reproductive health in your community? (probe for information on menstruation, unplanned pregnancy, STI, unprotected sex, HTS uptake etc.)
8. Also probe for the kind of information that young people get from each of the sources mentioned in question 10 (probe for information from father, mother, peers if not mentioned)
9. Which of these sources of information do you think are more effective?
10. What do young people do to protect themselves against STIs, unplanned pregnancy and HIV/AIDS in this community? (Probe for use of condom and where they get it, HTS and HIV care if not mentioned)
11. If there is an HIV positive young person in this community, what will be the reactions of the community members?
12. What do you think should be the roles of your ministry in reducing the spread of HIV in the community
13. Mention some reproductive health services that are not easily accessible to AYP? Probe for the barriers for accessing such services
14. What other information do you think may be useful for this study?
