# Supplementary material for: Combination prevention package of interventions for reducing vulnerability to HIV among adolescent girls and young women in Nigeria: An action research
Source: PLoS One. 2023 Jan 18;18(1):e0279077. doi: 10.1371/journal.pone.0279077 (PMC9847984; doi:10.1371/journal.pone.0279077)
Supplement: Attachment — Submitted filename: Plos one Review.docx [file pone.0279077.s004.docx]

**Review**

**Abstract**

The background section in the abstract reads as a method section. The background section should reflect studies that have examined similar interventions and the authors should discuss the gap in the literature and how a study like this fills that gap.

**Introduction**

The authors state that “Despite the myriad programs and agencies offering HIV-related interventions in the country”- The authors should mention 2-3 of these programs and describe the HIV-related interventions these programs offer in comparison to what is being proposed in this study.

The authors should either cite similar studies that have used action research methodology for HIV related research (in a different population, age group, or another disease) and mention how this study differs OR the authors should state that this will be the first study to their knowledge that uses the action methodology to address this burden.

Line 77 needs a citation – “The Breakthrough Series (BTS) collaborative lends itself to the ideals of action research.”

The authors define BTS well and state that the BTS approach has been used extensively but the authors fail to provide examples of studies that have used them. The authors can provide 1 or 2 examples in which the BTS method has been used

The last paragraph of this section should have the objective of the paper and the hypothesis. The authors should consider a revision.

**Methods**

The authors should clearly state the study design e.g. this is a qualitative study using xx grounded approach/methodology.

The authors state that “We purposively selected two local government areas (LGA) as implementation sites and one as the control site in each study location” but the authors fail to give a reason why?- The authors should consider providing a reason for this.

The authors mention “**local BTS teams** planned the specific interventions to address the change topics identified in each study state” But the authors do not report on who makes up these local BTS teams. The description of the teams needs to be reported first before the activities of these teams are described. The authors should consider describing these BTS teams and the type of stakeholders that constitute each of these teams.

A dedicated section for recruitment and population description needs to be included in the methods section i.e. how were youth participants and other local stakeholders recruited into the study. (please explain the process of recruitment). Describe all participants and stakeholders who participated in the study.

What was the duration of each action period (3 months? 4 months?). The actual duration needs to be stated as well as the timing (2019-2020?)

Line 59-60 needs to be revised “There were two action periods in this study, lasting three and two each.”- Do the authors mean to write 2- 3 months? This needs to be clarified

The section labeled household survey is reporting on demographics, which should be in the results section. The household survey should simply describe the methodology that was employed and should not report on results (including demographics) of the survey. E.g. line 69 *“The study population was females of the age range of 15 to 24 years old”* These errors are also seen in lines 193-195. All these should be in the results section, not the methods section.

The authors need to define acceptability (what do the authors hypothesize acceptability for this study will look like). If possible the authors should also cite their definition from the literature

The authors need to define accessibility (what do the authors hypothesize accessibility for this study will look like). If possible the authors should also cite their definition from the literature

**Results**

For each theme/domain idea, the authors should consider including quotes from participants in describing some of the results. This is only evident in the parental communication intervention themes onwards, previous themes like Peer-to-peer communication interventions and Youth facilitator-driven interventions do not have quotes. The authors should consider revising this to include quotes from stakeholders.

**Discussion**

The authors state that **“**Several authors have successfully implemented HIV prevention interventions based on the MPPI strategy among in-school and out-of-school adolescents, including community mobilization, outreach, advocacy, and monitoring”- But the authors do not describe some of these studies. The authors should consider describing 1 or 2 of these studies.

The authors should report on or cite other studies/programs from the literature (in other populations, countries, etc.) that have targeted AGYW and their parents for matters relating to sex or HIV prevention, etc. OR the authors must state that no such study exists in the literature and this study will be the first of its kind.

Line 445-446 needs a citation from the literature- “These buttress the point that one size will not fit all, and context is (nearly) everything in HIV prevention programming for AGYW.”

The authors must include a dedicated section in the discussion section to describe and discuss the study limitations. This should not be in the conclusion.

**Conclusion**

The conclusion section should simply summarize the overall findings and what future research should explore, the study limitations should be removed from this section and have a dedicated section in the discussion section.

**Overall Summary and reviewer final comments**

Overall this is a good paper, which takes an innovative approach to the topic. The authors should consider the revision suggestions above and also consider a clean copy edit of the manuscript as there are a few, run-on sentences, tense confusions, and grammatical errors which need to be fixed using spell check or software like Grammarly. Once these edits are incorporated this paper should be accepted for publication.
