# Supplementary material for: Combination prevention package of interventions for reducing vulnerability to HIV among adolescent girls and young women in Nigeria: An action research
Source: PLoS One. 2023 Jan 18;18(1):e0279077. doi: 10.1371/journal.pone.0279077 (PMC9847984; doi:10.1371/journal.pone.0279077)
Supplement: Attachment — Submitted filename: Response to Reviewers.docx [file pone.0279077.s005.docx]

PONE-D-21-01166 Minimum prevention package of interventions for reducing vulnerability to HIV among adolescent girls and young women in Nigeria: An action research

Dear Reviewers,

Thank you very much for the extensive review of our manuscript. We were very encouraged by your review. We have incorporated all the recommendations and suggestion in the uploaded revised version. Below is a point by point responses to the comments provided to us.

We look forward to finalizing the manuscript.

Thanks once again.

OAA

**Journal Requirements:**

1. Please ensure that your manuscript meets PLOS ONE's style requirements, including those for file naming. The PLOS ONE style templates can be found at 
https://journals.plos.org/plosone/s/file?id=wjVg/PLOSOne_formatting_sample_main_body.pdf and 
[https://journals.plos.org/plosone/s/file?id=ba62/PLOSOne_formatting_sample_title_authors_affiliations.pdf](https://journals.plos.org/plosone/s/file?id=ba62/PLOSOne_formatting_sample_title_authors_affiliations.pdf\)

Done

2. Please include additional information regarding the survey or questionnaire used in the study and ensure that you have provided sufficient details that others could replicate the analyses. For instance, if you developed a questionnaire as part of this study and it is not under a copyright more restrictive than CC-BY, please include a copy, in both the original language and English, as Supporting Information.  If the original language is written in non-Latin characters, for example Amharic, Chinese, or Korean, please use a file format that ensures these characters are visible.

The study questionnaire has been added as a supporting document

3. Please state whether you validated the questionnaire prior to testing on study participants. Please provide details regarding the validation group within the methods section.

The study tools were pretested before data collection to ensure face validity; however, we did not conduct a formal validation procedure.

4. Please include a copy of the interview guide used in the study, in both the original language and English, as Supporting Information, or include a citation if it has been published previously.

This has been included as a supporting document

5. You indicated that you had ethical approval for your study. In your Methods section, please ensure you have also stated whether you obtained consent from parents or guardians of the minors included in the study or whether the research ethics committee or IRB specifically waived the need for their consent.

A statement has been included on how informed consent was obtained

6. We note that the grant information you provided in the ‘Funding Information’ and ‘Financial Disclosure’ sections do not match. When you resubmit, please ensure that you provide the correct grant numbers for the awards you received for your study in the ‘Funding Information’ section.

If you would like to make changes to your financial disclosure, please include your updated statement in your cover letter.

This research was funded by the Global Fund to Fight AIDS, Tuberculosis and Malaria as part of its HIV New Funding Model. Olujide Arije was supported by the African Academy of Sciences (AAS) under a DELTAS Africa Initiative grant [107768/Z/15/Z] as part of the Consortium for Advanced Research Training in Africa (CARTA). CARTA is jointly funded by DELTAS, the Carnegie Corporation of New York [B 8606.R02], and Swedish International Development Cooperation Agency (SIDA) [54100029].

7. In your Data Availability statement, you have not specified where the minimal data set underlying the results described in your manuscript can be found. PLOS defines a study's minimal data set as the underlying data used to reach the conclusions drawn in the manuscript and any additional data required to replicate the reported study findings in their entirety. All PLOS journals require that the minimal data set be made fully available. For more information about our data policy, please see http://journals.plos.org/plosone/s/data-availability.

Upon re-submitting your revised manuscript, please upload your study’s minimal underlying data set as either Supporting Information files or to a stable, public repository and include the relevant URLs, DOIs, or accession numbers within your revised cover letter. For a list of acceptable repositories, please see http://journals.plos.org/plosone/s/data-availability#loc-recommended-repositories. Any potentially identifying patient information must be fully anonymized.

Important: If there are ethical or legal restrictions to sharing your data publicly, please explain these restrictions in detail. Please see our guidelines for more information on what we consider unacceptable restrictions to publicly sharing data: http://journals.plos.org/plosone/s/data-availability#loc-unacceptable-data-access-restrictions. Note that it is not acceptable for the authors to be the sole named individuals responsible for ensuring data access.

We will update your Data Availability statement to reflect the information you provide in your cover letter.

Minimal data set has been included

**Reviewer #1
Abstract**
The background section in the abstract reads as a method section. The background section should reflect studies that have examined similar interventions and the authors should discuss the gap in the literature and how a study like this fills that gap.

The abstract has been revised

Introduction
The authors state that “Despite the myriad programs and agencies offering HIV-related interventions in the country”- The authors should mention 2-3 of these programs and describe the HIV-related interventions these programs offer in comparison to what is being proposed in this study.

Some HIV-related interventions in the country have been mentioned

The authors should either cite similar studies that have used action research methodology for HIV related research (in a different population, age group, or another disease) and mention how this study differs OR the authors should state that this will be the first study to their knowledge that uses the action methodology to address this burden.

Some similar studies have been cited

Line 77 needs a citation – “The Breakthrough Series (BTS) collaborative lends itself to the ideals of action research.”

Citation has been added

The authors define BTS well and state that the BTS approach has been used extensively but the authors fail to provide examples of studies that have used them. The authors can provide 1 or 2 examples in which the BTS method has been used.

Some examples have been provided

The last paragraph of this section should have the objective of the paper and the hypothesis. The authors should consider a revision.

Objective of study has been added to the last paragraph

Methods
The authors should clearly state the study design e.g. this is a qualitative study using xx grounded approach/methodology.

The study design has been stated

The authors state that “We purposively selected two local government areas (LGA) as implementation sites and one as the control site in each study location” but the authors fail to give a reason why?- The authors should consider providing a reason for this.

Bases of selection of study locations have been stated

The authors mention “local BTS teams planned the specific interventions to address the change topics identified in each study state” But the authors do not report on who makes up these local BTS teams. The description of the teams needs to be reported first before the activities of these teams are described. The authors should consider describing these BTS teams and the type of stakeholders that constitute each of these teams.

Members of the BTS teams have been included.

A dedicated section for recruitment and population description needs to be included in the methods section i.e. how were youth participants and other local stakeholders recruited into the study. (please explain the process of recruitment). Describe all participants and stakeholders who participated in the study.

A dedicated section for recruitment section has been added

What was the duration of each action period (3 months? 4 months?). The actual duration needs to be stated as well as the timing (2019-2020?)

Duration information has been included

Line 59-60 needs to be revised “There were two action periods in this study, lasting three and two each.”- Do the authors mean to write 2- 3 months? This needs to be clarified

Clarification has been provided

The section labeled household survey is reporting on demographics, which should be in the results section. The household survey should simply describe the methodology that was employed and should not report on results (including demographics) of the survey. E.g. line 69 “The study population was females of the age range of 15 to 24 years old” These errors are also seen in lines 193-195. All these should be in the results section, not the methods section.

These errors have been corrected

The authors need to define acceptability (what do the authors hypothesize acceptability for this study will look like). If possible the authors should also cite their definition from the literature.

Acceptability has been defined

The authors need to define accessibility (what do the authors hypothesize accessibility for this study will look like). If possible the authors should also cite their definition from the literature

Accessibility has been defined


Results
For each theme/domain idea, the authors should consider including quotes from participants in describing some of the results. This is only evident in the parental communication intervention themes onwards, previous themes like Peer-to-peer communication interventions and Youth facilitator-driven interventions do not have quotes. The authors should consider revising this to include quotes from stakeholders.

The result section is divided into 2 part: ‘*The Change ideas’* and ‘*Reach and uptake of the change ideas’.* The first part was a brief narration of the various interventions (change ideas) that were developed and implemented across the different study locations. The second aspect described report findings from data collected form study participants. Kindly observe that all of the theme/domain ideas have quotes from participants in describing some of the results

Discussion
The authors state that “Several authors have successfully implemented HIV prevention interventions based on the MPPI strategy among in-school and out-of-school adolescents, including community mobilization, outreach, advocacy, and monitoring”- But the authors do not describe some of these studies. The authors should consider describing 1 or 2 of these studies.

Additional studies have been described

The authors should report on or cite other studies/programs from the literature (in other populations, countries, etc.) that have targeted AGYW and their parents for matters relating to sex or HIV prevention, etc. OR the authors must state that no such study exists in the literature and this study will be the first of its kind.

Additional studies/programs from the literature have been cited

Line 445-446 needs a citation from the literature- “These buttress the point that one size will not fit all, and context is (nearly) everything in HIV prevention programming for AGYW.”

A citation from the literature has been added

The authors must include a dedicated section in the discussion section to describe and discuss the study limitations. This should not be in the conclusion.

The study limitations have been included in the discussion section

Conclusion
The conclusion section should simply summarize the overall findings and what future research should explore, the study limitations should be removed from this section and have a dedicated section in the discussion section.

The conclusion has been modified accordingly

**Reviewer #2: Review**

However, my appreciation of the contribution of this research might be enhanced by a clearer presentation of methods, a more rigorous analysis of data (or at least a clear acknowledgment of its limitation), and a possible reframing of the discussion.

I found it difficult to follow the connection between action research, breakthrough series, a learning collaborative approach, and a plan-do-study-act cycle.

I also found it challenging to understand the relationship between change packages, the combination prevention intervention approach, intervention models, change ideas, and minimum prevention package of interventions.

The connection among the above concepts have been clarified especially under the ‘Implementation approach’, Learning Sessions and Action Session

Additionally, I believe it would be helpful to have additional information about what measures were evaluated when. For example,

- what was assessed during the "monitoring and evaluation component"?

This information has been included

- What did local CBOs evaluate to test change ideas?

The word test has been replaced with implement which we used interchangeably, and the role of the CBOs further clarified

- What criteria were used to determine "whether to adapt, adopt, abandon, increase scale, or test under different conditions?"

This information has been included

- What was measured at baseline and follow-up, and what was considered an "outcome of the specific interventions" and evaluated only at follow-up?

This information has been included

In terms of analysis, given that we don't know the baseline probability of receiving services, and the authors did not control for confounders or possible contamination effects, I'm wary of ascribing too much salience to the significant differences found between services used between the intervention LGAs and controls.

This detail has been included in the text as a limitation

Also, as multiple interventions were available in the LGAs, I believe the text would be improved by describing how authors ascertained that services used were due to a particular intervention.

The research assistants were trained to describe the intervention implemented in the different study locations to reduced misclassification bias. This information has been added

Finally, in the discussion, the authors state this their approach "helped improve the acceptability and accessibility of programs." I would be careful to overstate this point. While they've shown this approach results in acceptable and accessible programs, without comparing it to other models of intervention development and implementation, it's impossible to say whether it was an "improvement."

We have modified our statement to be more conservative.

However, I think the authors miss an opportunity to highlight how this collaborative approach, which engages the community and takes context into account, might be an improvement over other models. Consider framing the discussion to illuminate the relative benefits of this approach.

We have attempted to do highlight the collaborative approach
