# Supplementary material for: Combination prevention package of interventions for reducing vulnerability to HIV among adolescent girls and young women in Nigeria: An action research
Source: PLoS One. 2023 Jan 18;18(1):e0279077. doi: 10.1371/journal.pone.0279077 (PMC9847984; doi:10.1371/journal.pone.0279077)
Supplement: Attachment — Submitted filename: Reviewer recommendations.docx [file pone.0279077.s006.docx]

Thanks for your work on updating this paper and the presentation of an important, interesting study. I believe substantial work is needed to the methods sections (as well as other sections). There are a LOT of components of this study and it is going to be critical to make sure to be super specific in outlining each component and detailing how they all fit together. Implementation of these change ideas is a central component and I believe this paper could vastly benefit from incorporation of some implementation science framework and/or theory. Would also recommend a conceptual framework to outline how all the pieces fit together, the mechanism at play, and the implementation outcomes assessed. Highlighting how this work is embedded within the larger HIV response within Nigeria is also super important.

The abstract highlights that vulnerability and sociocultural contexts vary across Nigeria. Would expand upon this in the introduction and highlight some of these varying differences across the nation (also providing rationale to the selection of study locations).

The abstract notes that this study developed a minimum HIV prevention package for AGYW. How did this differ from package of care put forth within the Nigerian NPP 2010-2012 and the MPPI? Does the country currently have guidelines or recommendations for AYGW? How are these guidelines and recommendations similar and/or different from the FLHE curriculum or national Youth service corps program? These components feel disaggregated as they’re quite separated from one another within the introduction. Would recommend reframing.

The aim of the study put forth in the abstract differs slightly (from my understanding) from the aim of the study put forth in the last sentences of the introduction. Would recommend harmonizing.

It is helpful to see the citation of similar studies that have leveraged action research methods, per reviewer #1’s suggestion. Would recommend going a step further to outline the key lessons learned or implications for this work, ie connect it back to the ongoing study to see seems a bit more integrated and appropriate within the introduction.

In the methods it states “This research has a mixed-method design involving a cross-sectional descriptive study, focus group discussions with AGYW, and one-on-one interviews with selected key informants.” I find this a tad confusing. Specifically, can you specify which methods are quantitative verse qualitative? Are the one-on-one interviews, in-depth qualitative interviews?

Great to see you’ve incorporated justification for study site selection. Would be helpful to outline in the introduction the differences and distribution of HIV prevalence among AGYW across states of Nigeria.

In lines 153-156 it states that interventions were selected based on estimated youth population and the absence of existing interventions. Were there prior studies conducted to provide size estimations of AGYW in these areas? Please specify. Also, what was done previously to document and quantify existing interventions within these areas? Please specify. How recently were these things done?

You define a comprehensive change package. But how do you define “desired change.” Would recommend looking at specific implementation outcomes to quantify.

Impressive how collaborative teams were across levels and programs. Would you add a few sentences to outline how communication occurred across team and how consistency in implementation was guaranteed?

Who conducted the scoring of the change topics? How was this process conducted and how was the prioritization matrix utilized? Was there 1 matrix overall or per study site? Did this happen once or iteratively? Were AGYW involved in the process?

It’s noted that the third learning session was to review the implementation process. How was this done? How was implementation planned and monitored?

You outline that location CBOs were engaged in the implementation of the change ideas. How were CBOs trained? How was uniform implementation ensured? How was fidelity of the implementation assessed? Did all CBOs have similar staffing and resources?

Can you specify what you mean by a “A multi-stage sampling technique was employed to sample participants at enumeration area-level and at household level.” – I am struggling to understand. Was the sampling the same for baseline and endline?

What does “Although the overall evaluation of the action research involved a baseline and end-line assessment, the outcome of specific interventions was accessed only at the end-line since those interventions were not already designed at the start of the research and would not fit a pre-post assessment” mean? How was engagement in HIV prevention services assess at the baseline survey?

Were respondents reimbursed for participation in the survey? Were participants reimbursed for qualitative interviews?

What was the sample size for the endline survey?

Were qualitative interviews conducted with AGYW who participated in the interventions specifically? All interventions or some? How was sampling done for the qualitative interviews? What was the sample size? There is mention of snowball sampling within the results but it is unclear for which component and this level of detail should be included within the methods.

How was intervention uptake quantified? How was the denominator determined?

Were all qualitative interviews coded with the same codebook? How was coding discrepancies resolved among the 4 coders? Were interview singly or doubly coded?

Was there any additional eligibility criteria for participants? What about if AGYW were living with HIV, were they included? What was the eligibility criteria for key informants?
