# Supplementary material for: Combination prevention package of interventions for reducing vulnerability to HIV among adolescent girls and young women in Nigeria: An action research
Source: PLoS One. 2023 Jan 18;18(1):e0279077. doi: 10.1371/journal.pone.0279077 (PMC9847984; doi:10.1371/journal.pone.0279077)
Supplement: Attachment — Submitted filename: Manuscript Review.docx [file pone.0279077.s007.docx]

Dear Editor,

Thank you so much for giving me an opportunity to review the manuscript. First, I had to read comments of earlier reviewers. The authors have addressed most concerns raised by earlier reviewers. I make the following comments:

Title: The authors might consider revising the title to include or replace ‘minimum’ with ‘combined’ to tie with the objective of the study. In addition, the authors might consider including adolescent boys and young men in the title because results show that these people participated in the study too.

Abstract: Lines 22-25 read like methods section. Authors should consider including recommendation(s) [it can be within the conclusion]. Proofread for typo errors e.g. Line 19 ‘adolescents’ ‘[adolescent]’.

Introduction: First statement needs revision – whether it is about adolescent girls or adolescents in general. The section needs proofreading for grammatical errors. Transitions are lacking between some paragraphs.

Methods and Materials: Lines 217-218 need citation(s).

Results: Presentation of results is not clear especially at the beginning. I suggest focusing on the results and removing some background information. Some information might be used to strengthen the Methods and Materials section.

Proofread for typo, grammar and spelling errors e.g. Line 423, line 458, line 497, line 533, line 545 etc.

It is only when I get to line 479 that I begin reading of young men. Again, these are not reflected in the title, abstract, introduction, methods and materials sections. Provide an example to lines 484-487 or cite the literature. Some quotes are not identified (have no persons who said them). Other quotes can be shortened.

Discussion and Conclusion sections are clear to me.

Reference section needs editing for consistency including for dates.

Others: Check whether it is HIV/AIDS or [HIV and AIDS]

Thank you.
