# Supplementary material for: Combination prevention package of interventions for reducing vulnerability to HIV among adolescent girls and young women in Nigeria: An action research
Source: PLoS One. 2023 Jan 18;18(1):e0279077. doi: 10.1371/journal.pone.0279077 (PMC9847984; doi:10.1371/journal.pone.0279077)
Supplement: Attachment — Submitted filename: Response.docx [file pone.0279077.s008.docx]

**Reviewer #3**:

General comments

I don’t see discussion of or reflection on diversity in sexuality. Was there reflection on heteronormativity in socio-cultural contexts, and how this was embedded in parent-adolescent sexuality communication?

- How does the centrality and positioning of the church as a mechanism through which sexuality communication and education occurs shape the content and framing of sex? E.g. discussions of sexual pleasure and empowerment versus ‘abstinence until marriage’ etc? It would be good to see some reflection on this.

- Norms around sexuality communication are mentioned – e.g. appropriateness of discussing sexual organs/genitalia, and euphemistic nature of language prohibiting open and explicit discussion – how does this impact young people’s knowledge and sexual decision making?

- There is evidence of parental resistance to comprehensive sexuality education in this context (CSE)? Was this also the case at a broader community and institutional level (e.g schools, churches)? How does this impact intervention delivery?

All interventions in our study were developed, tested and refined iteratively through co-creation with AGYW as well as other stakeholders in each local study site. These interventions were contextual and we didn’t find substantial evidence of parental resistance to engaging their children/wards on sexual education particularly in the intervention locations, or of AGYW resistance in receiving sexual education from their parents as documented in our findings. Moreover we were careful to allow the community members have substantial inputs in order not to impose the world view of the researchers on them. What we have reported is the findings using the action research approach findings from assessing exposure to the interventions implemented. These interventions were done in very limited scale but future studies can help to explore broader context and implication to parental engagement in the sexuality of the adolescents and young people. It is important for us as researchers to continue to be culturally sensitive, and not to foist what might be interpreted as western ideas on non-western local communities given that different societies are at different levels or phases of social progressiveness.

Specific comments:

Page 2

- Line 19: re-order sentence – “AGYW in Nigeria…”

This has been corrected

Page 5

- Line 100: why is “problem’s” possessive? Incorrect use of apostrophe

This has been corrected

Page 7

- Lines 134-137: check brackets are closed

This has been corrected

Page 17

- Line 370: surely a 21 year old female is a “young woman”, not a “girl”? Refer to standard definitions of “girls” vs “women”, and age brackets of AGYW

This has been corrected

**Reviewer #4**

Thanks for your work on updating this paper and the presentation of an important, interesting study. I believe substantial work is needed to the methods sections (as well as other sections). There are a LOT of components of this study and it is going to be critical to make sure to be super specific in outlining each component and detailing how they all fit together.

- Implementation of these change ideas is a central component and I believe this paper could vastly benefit from incorporation of some implementation science framework and/or theory.

We consider this suggestion important but we are weary of adopting an implementation research framework since that was not how the intervention was carried out. We prefer to stay fully within an action research context which we consider as the proper way to view and report the research.

- Would also recommend a conceptual framework to outline how all the pieces fit together, the mechanism at play, and the implementation outcomes assessed.

We have included a conceptual framework

- Highlighting how this work is embedded within the larger HIV response within Nigeria is also super important.

We have included a statement to highlight this

- The abstract highlights that vulnerability and sociocultural contexts vary across Nigeria. Would expand upon this in the introduction and highlight some of these varying differences across the nation (also providing rationale to the selection of study locations).

We provided rationale for selection of the study locations under ‘Study setting’.

- The abstract notes that this study developed a minimum HIV prevention package for AGYW. How did this differ from package of care put forth within the Nigerian NPP 2010-2012 and the MPPI? Does the country currently have guidelines or recommendations for AYGW? How are these guidelines and recommendations similar and/or different from the FLHE curriculum or national Youth service corps program? These components feel disaggregated as they’re quite separated from one another within the introduction. Would recommend reframing.

The country is still in the process of developing a scalable guide for the implementation of community-based HIV programs focused on AYP in Nigeria (personal communication). The package of care put forth in the Nigerian NPP 2010-2012 and the MPPI are generic while the prevention package we present in this study are adaptive and contextualized. The FLHE curriculum and National Youth Service Corps peer-educator program are earlier approaches to scale-up HIV prevention and are not necessarily combination prevention approaches.

- The aim of the study put forth in the abstract differs slightly (from my understanding) from the aim of the study put forth in the last sentences of the introduction. Would recommend harmonizing.

We have revised this

- It is helpful to see the citation of similar studies that have leveraged action research methods, per reviewer #1’s suggestion. Would recommend going a step further to outline the key lessons learned or implications for this work, ie connect it back to the ongoing study to see seems a bit more integrated and appropriate within the introduction.

We have further revised this section

- In the methods it states “This research has a mixed-method design involving a cross-sectional descriptive study, focus group discussions with AGYW, and one-on-one interviews with selected key informants.” I find this a tad confusing. Specifically, can you specify which methods are quantitative verse qualitative? Are the one-on-one interviews, in-depth qualitative interviews?

This has been revised

- Great to see you’ve incorporated justification for study site selection. Would be helpful to outline in the introduction the differences and distribution of HIV prevalence among AGYW across states of Nigeria.

We have updated the information in the “Study setting’ section

- In lines 153-156 it states that interventions were selected based on estimated youth population and the absence of existing interventions. Were there prior studies conducted to provide size estimations of AGYW in these areas? Please specify. Also, what was done previously to document and quantify existing interventions within these areas? Please specify. How recently were these things done?

This has been revised

- You define a comprehensive change package. But how do you define “desired change.” Would recommend looking at specific implementation outcomes to quantify.

Desired change was contextual for each ‘change idea’ implemented and was evaluated using the decision rule for implemented change ideas (Table 3 in the manuscript)

- Impressive how collaborative teams were across levels and programs. Would you add a few sentences to outline how communication occurred across team and how consistency in implementation was guaranteed?

We have introduced a chart to show the line of communication

- Who conducted the scoring of the change topics? How was this process conducted and how was the prioritization matrix utilized? Was there 1 matrix overall or per study site? Did this happen once or iteratively? Were AGYW involved in the process?

Scoring of the change topics using the prioritization matrix was conducted during the learning sessions which held separately in each study location when the local BTS needed to decide on which change topic to intervene for. AGYW were members of the local BTS team (stated earlier under section titled ‘BTS Collaborative management’.

- It’s noted that the third learning session was to review the implementation process. How was this done? How was implementation planned and monitored?

We used the monitoring and evaluation reports from each of the interventions implemented. We have revised this section in the manuscript

- You outline that location CBOs were engaged in the implementation of the change ideas. How were CBOs trained? How was uniform implementation ensured? How was fidelity of the implementation assessed? Did all CBOs have similar staffing and resources?

Because of the diversity of interventions across the different study locations, the CBOs engaged did not have similar staffing and resources. Even then, it was not necessary to have uniformity across the study locations since different interventions were implemented in different places. CBOs were engaged according to the need in each study site and the research provided site-specific training and support as was necessary for the CBOs that were engaged

- Can you specify what you mean by a “A multi-stage sampling technique was employed to sample participants at enumeration area-level and at household level.” – I am struggling to understand. Was the sampling the same for baseline and endline?

The sampling method was the same at baseline and at endline but it was not a panel data. Different set of respondents were recruited from the same locations at the different times although a few participated in both the baseline and endline survey (but not be design). We have refrained from dwelling on the methodology of the baseline assessment since we did not report it in this current report.

- What does “Although the overall evaluation of the action research involved a baseline and end-line assessment, the outcome of specific interventions was accessed only at the end-line since those interventions were not already designed at the start of the research and would not fit a pre-post assessment” mean? How was engagement in HIV prevention services assess at the baseline survey?

The action research design we adopted does not fit a pre-post assessment because the specific interventions implemented were designed after there was a baseline assessment. Findings from the baseline assessment were part of the sources of information for designing the various intervention. Because these interventions were non-exist as at baseline, no specifc data can be collected about them at baseline. In this report, we did not include any baseline data. We have revised the section to more properly reflect what we did.

- Were respondents reimbursed for participation in the survey? Were participants reimbursed for qualitative interviews?

All FGD and IDI participants received transportation reimbursement of ₦1000 each ($1≈₦360, 2017) Key informants were not reimbursed because interviews took place in their various offices.

- What was the sample size for the endline survey?

The total sample size was 4308, and we have updated the manuscript in this respect

- Were qualitative interviews conducted with AGYW who participated in the interventions specifically? All interventions or some? How was sampling done for the qualitative interviews? What was the sample size? There is mention of snowball sampling within the results but it is unclear for which component and this level of detail should be included within the methods.

We have updated this section to include the various categories of participants in the FGD. We included both participants and non-participants of the action research implementation component in the FGD. Snowballing was used as part of the intervention component not evaluation component of the action research. We have moved the section describing the ‘Change ideas’ to the methodology.

- How was intervention uptake quantified? How was the denominator determined?

The intervention uptake was quantified as a proportion of participants that reported exposure to the various groups of interventions. The denominator in each study state was the total number of respondents in each state

- Were all qualitative interviews coded with the same codebook? How was coding discrepancies resolved among the 4 coders? Were interview singly or doubly coded?

A generic codebook was used across all transcripts but adapted to suit different groups of study participants. We have also updated this session

- Was there any additional eligibility criteria for participants? What about if AGYW were living with HIV, were they included? What was the eligibility criteria for key informants?

We have revised this section

**Reviewer #5**

Thank you so much for giving me an opportunity to review the manuscript. First, I had to read comments of earlier reviewers. The authors have addressed most concerns raised by earlier reviewers. I make the following comments:

Title: The authors might consider revising the title to include or replace ‘minimum’ with ‘combined’ to tie with the objective of the study. In addition, the authors might consider including adolescent boys and young men in the title because results show that these people participated in the study too.

We agree with your suggestion to replace minimum with combination. However, our interventions targeted AGYW, and boys and young men were only included in the study as stakeholders in the vulnerability of AGYW to HIV, Hence, we consider it more appropriate to retain the AGYW focus

Abstract: Lines 22-25 read like methods section. Authors should consider including recommendation(s) [it can be within the conclusion]. Proofread for typo errors e.g. Line 19 ‘adolescents’ ‘[adolescent]’.

We have revised this

Introduction: First statement needs revision – whether it is about adolescent girls or adolescents in general. The section needs proofreading for grammatical errors. Transitions are lacking between some paragraphs.

We have revised this

Methods and Materials: Lines 217-218 need citation(s).

We have revised this

Results: Presentation of results is not clear especially at the beginning. I suggest focusing on the results and removing some background information. Some information might be used to strengthen the Methods and Materials section.

We have moved the section describing the change ideas to the methodology

Proofread for typo, grammar and spelling errors e.g. Line 423, line 458, line 497, line 533, line 545 etc.

We have revised this

It is only when I get to line 479 that I begin reading of young men. Again, these are not reflected in the title, abstract, introduction, methods and materials sections. Provide an example to lines 484-487 or cite the literature. Some quotes are not identified (have no persons who said them). Other quotes can be shortened.

The interventions we have described in our manuscript are focused on AGYW. Young men were included in the action research only as stakeholders like other stakeholders such as the gatekeepers (religious and community leaders) and key informants (health workers, program officers etc).

Discussion and Conclusion sections are clear to me.

Reference section needs editing for consistency including for dates.

We have revised the reference section

Others: Check whether it is HIV/AIDS or [HIV and AIDS]

We have updated this to HIV and AIDS

Thank you.
