# Supplementary material for: Combination prevention package of interventions for reducing vulnerability to HIV among adolescent girls and young women in Nigeria: An action research
Source: PLoS One. 2023 Jan 18;18(1):e0279077. doi: 10.1371/journal.pone.0279077 (PMC9847984; doi:10.1371/journal.pone.0279077)
Supplement: Attachment — Submitted filename: Response to reviewers.docx [file pone.0279077.s009.docx]

**Response to reviewers**

1. Line 163: “Combination HIV prevention is the recommended approach for comprehensive HIV prevention.” – does this sentence have value? Would benefit from being rephrased.

‘Combination HIV prevention’ is a specific terminology in HIV prevention and care. However we have revised this line to provide more clarity without loosing meaning.

2. Line 192: conceptual framework “used by / proposed by” Chimbindi et al?

This line has been corrected

3. Can the authors make more specific and actionable recommendations based on their findings? For example, strategies for supporting parent-adolescent SRH communication.
4. How much do parents’ own communication skills and SRH knowledge play a role? How can interventions bolster parents’ communication skills – such as those in the Akers paper the author cites. Also in: DiIorio et. (2000). Social cognitive factors associated with mother-adolescent communication about sex. Journal of Health Communication, 5(1), 41–51. doi:10.1080/108107300126740
5. How does parents’ own self-efficacy play a role? How can interventions best provide parents/caregivers with a knowledge base and skill-set, to enhance their motivation and confidence in communicating around SRH topics?
   See discussion on this in:
   a. - Guilamo-Ramos et al. (2008). Parent-adolescent communication about sexual inter- course: An analysis of maternal reluctance to communicate. Health Psychology, 27(6), 760–769. doi:10.1037/a0013833
   b. - Seif et al. (2018). Caretaker-adolescent communication on sexual and reproductive health: A cross- sectional study in Unguja-Tanzania Zanzibar. BMC Public Health, 18(31), 1–13. doi:10.1186/ s12889-017-4591-2
6. How can interventions empower parent/caregivers in order to increase their ability to be responsive to the needs of adolescents and support them to make safe and informed decisions about sex? Can the authors make recommendations pertaining to this issue.
   See discussion on this in:
   a. - Grossman et al. (2017). “We talked about sex.” “No, we didn’t”: Exploring adolescent and parent agreement about sexuality communication. American Journal of Sexuality Education. doi:10. 1080/15546128.2017.1372829
   b. - Nilsson et al. (2020). Obstacles to intergenerational communication in care- givers’ narratives regarding young people’s sexual and reproductive health and lifestyle in rural South Africa. BMC Public Health, 20(791). doi:10.1186/s12889-020-08780-9
7. If socio-cultural norms impede open sexuality communication, is there a strategy for helping parents/elders to understand the implications of their attitudes and communication style on adolescent sexual decision making? Specific recommendations relating to this issue would be helpful. See discussion of this in:
   a. - AVAC. (2018). Breaking the cycle of transmission: Increasing adoption of and adherence to effective HIV prevention among high-risk adolescent girls and young women.
   b. - Duby et al. (2022) ‘I can't go to her when I have a problem’: sexuality communication between South African adolescent girls and young women and their mothers, SAHARA-J: Journal of Social Aspects of HIV/AIDS, 19:1, 8-21, <https://doi.org/10.1080/17290376.2022.2060295>
8. How can policy makers and programme designers ensure that interventions are designed and implemented in ways that are sensitive to local cultural and linguistic norms and beliefs? See the Nilson and Duby references cited above for discussion of this. Including recommendations to consider this would be beneficial.

We have address the issues raised in Nos. 3-8 above with an additional paragraph as follows:

Concerning parent-adolescent SRH communication, interventions that are likely to work are those that specifically train parents in soft communications skills such as talking less, listening more, being less judgmental, and asking more questions in interactions with adolescents about SRH [44]. Efforts should be directed at boosting the self-efficacy of parents (i.e. confidence in the ability to discuss sexual issues with their adolescent) by increasing their knowledge about adolescent SRH [45,46]. Also, interventions that will increase the motivation of parents to communicate with their adolescents, including both males and females, as well as on all appropriate SRH topics are likely to be effective [47]. An important consideration for program design is to create programs that target parent/caregiver-adolescent pairs as there is some evidence that they are effective [48]. The interventions that mediate the intergenerational gaps between parents and adolescents by finding a common ground for them are likely to be effective interventions as such gaps have been implicated in poor parent-adolescent communications [49]. Some interventions may be presented via traditional and social media in order to bring them to scale. For example, the use of radio drama has a good history of success in social behavior communication change [50,51]. Socio-cultural norms impede open sexuality communication, and it is a major impedance to parent-adolescent communication about SRH. Yet, interventions must be sensitive to local cultural and linguistic norms and beliefs for them to gain any traction. Adopting co-creation approaches from the ground up with the people for whom the interventions are targeted, as we did in this study, can help to navigate the norms that are peculiar to different groups.
